# EP1 receptor within the ventrolateral periaqueductal grey controls thermonociception and rostral ventromedial medulla cell activity in healthy and neuropathic rat

**DOI:** 10.1186/1744-8069-7-82

**Published:** 2011-10-24

**Authors:** Enza Palazzo, Francesca Guida, Luisa Gatta, Livio Luongo, Serena Boccella, Giulia Bellini, Ida Marabese, Vito de Novellis, Francesca Rossi, Sabatino Maione

**Affiliations:** 1 Department of Experimental Medicine, Pharmacology Division, The Second University of Naples, via Costantinopoli 16, 80138 Naples, Italy; 2 Department of Pediatrics, The Second University of Naples, via De Crecchio 4, 80138 Naples, Italy

**Keywords:** EP1 receptor, tail flick, ON and OFF cell activity, antinociceptive descending pathway, spared nerve injury, rat.

## Abstract

The aim of this study was to investigate the expression of prostaglandin EP1 receptor within the ventrolateral periaqueductal grey (VL PAG). The role of VL PAG EP1 receptor in controlling thermonociception and rostral ventromedial medulla (RVM) activity in healthy and neuropathic rats was also examined. EP1 receptor was indeed found to be expressed within the VL PAG and co-localized with vesicular GABA transporter. Intra-VL PAG microinjection of ONO-DI-004, a selective EP1 receptor agonist, dose-dependently reduced tail flick latency as well as respectively increasing and decreasing the spontaneous activity of ON and OFF cells. Furthermore, it increased the ON cell burst and OFF cell pause. Intra-VL PAG prostaglandin E2 (PGE2) behaved similarly to ONO-DI-004. The effects of ONO-DI-004 and PGE2 were antagonized by intra-VL PAG L335677, a selective EP1 receptor antagonist. L335677 dose-dependently increased the tail flick latency and ongoing activity of the OFF cells, while reducing the ongoing ON cell activity. It also decreased the ON cell burst and OFF cell pause. In neuropathic rats using spare nerve injury (SNI) of the sciatic nerve model, EP1 receptor expression decreased in the VL PAG. However, ONO-DI-004 and L335677 were able to alter pain responses and ON and OFF cell activity, as they did in healthy animals. Collectively, these data show that within the VL PAG, EP1 receptor has a facilitatory effect on the nociceptive response and consistently affects RVM neuron activity. Thus, the blockade of EP1 receptor in the VL PAG leads to antinociception in neuropathic pain conditions, despite its down-regulation. The expression of EP1 receptor on GABAergic neurons is consistent with an EP1 receptor blockade-induced disinhibition of the antinociceptive descending pathway at VL PAG level.

## Background

It has been well established that prostaglandin E2 (PGE2) sensitizes peripheral nociceptors through the activation of prostaglandin EP receptors present on the peripheral terminals of sensory neurons, leading to a reduction in pain threshold and increased responsiveness [1]. As well as a peripheral role, spinal prostaglandins (PGs) contribute to dorsal horn sensitization in persistent pain states in the spinal cord [2,3]. Nevertheless, little attention has been given to PG action at supraspinal level and in particular within the antinociceptive descending pathway, consisting of periaqueductal grey (PAG), rostral ventromedial medulla (RVM) and spinal dorsal horn components. PAG-induced control of nociception is produced concomitantly with the modulation of neuron activity within the RVM: ON-cells, which are activated and OFF-cells, which are inhibited by **cutaneous **nociceptive stimuli [4]. Unlike ON and OFF cells, another class of neurons; the neutral cells, are instead unaffected by noxious stimuli.

Both isoforms of cyclooxygenases (COXs), COX-1 and COX-2, PGE2 and the prostaglandin EP3 receptor have been identified within the PAG [5-7]. Intra-PAG microinjection of a COX1-2 inhibitor, lysine-acetylsalcylate, reduced nociceptive processing [8,9]. The involvement of PAG PGs in tonic facilitatory control on spinal nociception [10] and that of PGE2 in the genesis of hyperalgesia and spontaneous pain at spinal dorsal horn level [11] has already been recognized. On this subject, EP receptor subtype antagonist may potentially behave like an analgesic at this level. Indeed, in one of our previous studies, we demonstrated that intra-PAG microinjections of EP1-4 receptor subtype antagonists prevented formalin and misoprostol-induced hyperalgesia in mice, demonstrating a key role of PGs in a facilitating nociceptive response throughout EP receptors at PAG level [12]. As far as chronic pain is concerned, insights into the role of EP1 receptor are still scant, although it has been reported that selective pharmacological blockade of EP1 receptor or its gene ablation counteracts pain in animal models of neuropathic pain [13-17]. Stimulation of EP1 receptor leads to a [Ca^2+^] increase and neurotransmitter release [3,18], and its pharmacological manipulation within the descending pathway of pain could therefore be a suitable strategy for pain relief. Since there is no evidence to date of EP1 receptor expression in the VL PAG, in this study we investigated the presence of EP1 receptor within the VL-PAG, its possible contribution to thermonociception and to the modulation of the ongoing and tail flick-related activity of RVM ON and OFF cells in physiological and neuropathic pain conditions.

## Methods

### Animals

Male Wistar rats (220-250 g) were housed under controlled conditions (12 h light/12 h dark cycle; temperature 20-22°C; humidity 55-60%) with chow and tap water available ad libitum. All surgery and experimental procedures were performed during the light cycle and were approved by the Animal Ethics Committee of The Second University of Naples. Animal care was in compliance with Italian (D.L. 116/92) and EC (O.J. of E.C. L358/1 18/12/86) regulations on the protection of laboratory animals. All efforts were made to reduce both animal numbers and suffering during the experiments.

### The spared nerve injury **model**

The spared nerve injury model of neuropathic pain was induced according to the method used by Decosterd and Woolf [19]. Rats were anaesthetised with sodium pentobarbital (50 mg/kg, i.p.). The sciatic nerve was exposed at the level of its trifurcation into sural, tibial and common peroneal nerves. The sural and common peroneal nerves were ligated tightly then transected just distal to the ligation, leaving the tibial nerve intact. Sham rats were anaesthetised, the sciatic nerve was exposed at the same level, but not ligated. Seven days after surgery sham and SNI rats underwent tail flick tests coupled with single unit extracellular recording experiments or were sacrificed for immunohistochemistry, RT-PCR and western blot analysis.

### Immunohistochemistry

Healthy, sham and SNI rats where anaesthetized with pentobarbital (50 mg/kg, i.p.) and transcardially perfused with saline solution followed by 4% paraformaldehyde in 0.1 M phosphate buffer. The brain was removed, post fixed for 4 hours in the perfusion fixative, cryoprotected for 72 hours in 30% sucrose in 0.1 M phosphate buffer and frozen in O.C.T. embedding compound. Transverse sections (15 μm) were cut using a cryostat and those containing the whole PAG were thaw-mounted onto glass slides. Sections were subsequently incubated for 1 day at room temperature in a humid chamber with the respective polyclonal antibodies (all diluted in block solution). All sections were processed for rabbit anti-vesicular glutamate transporter-1 (VGluT1) (1: 500, SySy, Germany), rabbit anti-vesicular GABA transporter (VGAT) (1:250, SySy, Germany) and goat-anti EP1 receptor (1:100, Santa Cruz, USA).

Following incubation, sections were washed and incubated for 3 hours with secondary antibody solution (goat anti-rabbit, or donkey anti-goat, IgG-conjugated Alexa FluorTM 488 and 568; 1:1000; Molecular Probes, USA). Slides were washed, cover-slipped with Vectashield mounting medium (Vector Laboratories, USA) and visualized under a Leica fluorescence microscope.

**Figure 1:**
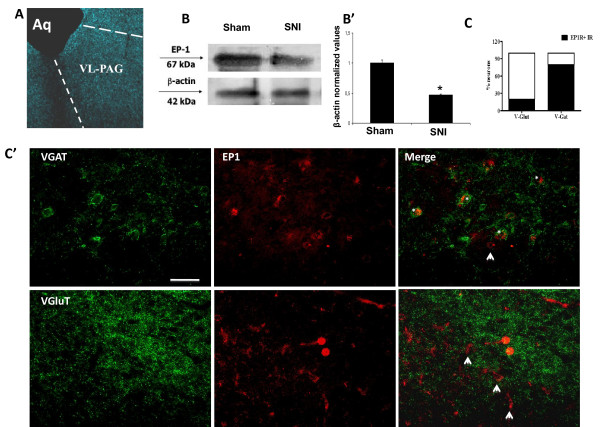
***A *shows a high magnification microscopy microphotography of VL PAG**. *B *and *B' *show the western blot analysis and relative quantification of EP1 receptor protein levels within the VL PAG in sham and SNI rats. *C *shows the double staining quantification for GABA vesicular transporter (VGAT) and glutamate vesicular transporter (VGluT) with EP1 receptor. The merge in *C' *indicates the co-localization of EP1 (red) and VGAT (green) labelled profiles. The arrow in the upper panel represents a positive EP1 profile which is not counterstained with VGAT, while the asterisks represent the double positive labelling EP1/VGAT. The lower panel shows the double staining for vesicular glutamate transporter VGluT1 (green) and EP1 receptor (red). The merge indicates the extremely scarce presence of EP1 in the VGluT1 labelled glutamatergic neuron, as highlighted by the arrows. Data represent mean ± S.E.M., **n = 6 **rats per group. Scale bars = 50 μm.

Quantitative analysis of EP1/VGluT1 and EP1/VGAT co-localization was performed within the ventrolateral sub region of the PAG (Figure 1A) by an observer blind to the experiment. Only the stained cells counterstained with DAPI were counted as positive profiles. Negative control by using secondary antibodies alone did not reveal any positive staining.

### Western blotting

A block of tissue containing the PAG was cut using a vibrotome (Vibratome 1500, Warner Instruments, Hamden, CT, USA). A brainstem slice of 1.5 mm was cut throughout the rostral part of the PAG (interaural from +1.7 mm to +0.2 mm) [20]and the VL sub region was then isolated under optical microscope (M650, Wild Heerbrugg, Switzerland). VL PAG was first minced into small pieces with a blender, and then incubated overnight at 4°C with goat polyclonal anti-EP1 antibody (1:200 dilution; sc-22648, Santa Cruz, CA, USA); reactive bands were detected by chemiluminescence (SuperSignal, West Femto, Pierce, USA) on a ChemiDoc station (BioRad). Thirty micrograms of protein derived from 6 pooled VL PAG tissues were loaded for each sample. An anti-beta-actin (1:5000; Sigma, Milan, Italy) was used to check for identical protein loading. Images were captured using a ChemiDoc XRS system (Biorad), stored, and analyzed with the Quantity One software (BioRad). The quantified values were normalized to beta-actin, which was chosen as housekeeping protein.

### Surgical preparation for single unit electrophysiological recordings coupled with tail flick

For electrophysiological experiments combined with the tail flick test, rats were anaesthetised with pentobarbital (50 mg/kg, i.p.) and a 26-gauge, 12 mm long stainless steel guide cannula was stereotaxically lowered until its tip was 1.5 mm above the left VL PAG by applying coordinates (A: -7.8 mm from bregma, L: 0.5 mm, V: 4.5 mm below the dura) from the Atlas of Paxinos and Watson [20]. The cannula was anchored with dental cement to a stainless steel screw in the skull. David Kopf stereotaxic apparatus was used (David Kopf Instruments, Tujunga, CA, USA) with the animal positioned on a homeothermic temperature control blanket (Harvard Apparatus Limited, Edenbridge, Kent) set at 37°C. Surgery for cannula implantation was carried out the same day as the electrophysiology coupled with tail flick experiments.

### Intra-VL PAG microinjections

Direct intra-VL PAG administration of drugs, or respective vehicle, was conducted with a stainless steel cannula connected to a SGE 1-microlitre syringe via a polyethylene tube, inserted through the guide cannula and extended 1.5 mm beyond its tip to reach the VL PAG. Vehicle or drug solutions were administered into the VL PAG in a final volume of 200 nl. Microinjection was performed over a period of 60 sec and the injection cannula gently removed 2 min later. At the end of the experiment, a volume of 200 nl of neutral red (0.1%) was also injected into the VL PAG 30-40 min prior to killing the rat. Rats were then perfused intracardially with 20 ml phosphate buffer solution (PBS) followed by 200 ml 10% formalin solution in PBS. The brains were removed and immersed in a saturated formalin solution for 2 days. The injection sites were ascertained by using 2 consecutive sections (40 μm), one stained with neutral red to identify nuclei and the other unstained in order to determine dye spreading.

### RVM extracellular recordings

After implantation of the guide cannula into the VL PAG, a glass insulated tungsten filament electrode (3-5 MΩ) (Frederick Haer & Co., ME, USA) was stereotaxically lowered through a small craniotomy into the RVM (2.8-3.3 mm caudal to lambda, 0.4-0.9 mm lateral and 8.9-10.7 mm depth from the surface of the brain), using the coordinates from the atlas of Paxinos and Watson [20]. The jugular vein was cannulated so as to facilitate the intravenous administration of the anaesthetic (propofol, 8-10 mg/kg/h, i.v.). Anaesthesia was adjusted so that tail flicks were elicited with a constant latency < 6 sec. The level of anaesthesia was considered stable if animals maintained a constant tail flick latency lasting 30-40 min before recording. We did not observe any overt changes in latency in reaching anaesthetic stability in SNI, sham and healthy rats, although SNI rats showed a lower tail flick latency than controls. The thermal stimulus was elicited by a radiant heat source of a tail flick unit (Ugo Basile, Varese, Italy) focused on the rat's tail approximately 4-5 cm from its tip. The tail was placed over the surface of a slightly projecting window receiving the I.R. energy. The I.R. intensity of the tail flick unit in our experiments was set to 50 mW corresponding to 50 mJ per sec. Tail flick latency in seconds was determined by a timer connected to a photoelectric cell which stopped the timer (and switched off the lamp) whenever the the tail was withdrawn. Tail flicks were elicited every 5 min for at least 15 min prior to microinjecting drugs, or the respective vehicle, 0.05% dimethyl sulfoxide (DMSO) in artificial cerebrospinal fluid (ACSF, composition in mM: KCl 2.5; NaCl 125; MgCl_2 _1.18; CaCl_2 _1.26) into the VL PAG. Spike waveforms were displayed on an oscilloscope in order to ensure that the unit under study was unambiguously discriminated throughout the experiment. Signals were also processed using an interface (CED 1401) (Cambridge Electronic Design Ltd., UK) connected to a Pentium III PC. Spike2 software (CED, version 4) was used to create peristimulus rate histograms on-line and to store and analyse digital records of single-unit activity off-line. Configuration, shape, and height of the recorded action potentials were monitored and recorded continuously using a window discriminator and Spike2 software for on-line and off-line analysis. Once background cellular activity was detected during cell search within the RVM, ON and OFF cells were identified by the tail flick stimulus. ON cells were identified by a burst of activity beginning immediately prior to a nocifensor reflex to the tail flick, while OFF cells were identified by the fact that they ceased firing at that time [21]. Three or more thermal noxious stimulus trials were applied through the tail flick unit to the tail at intervals of 5 min in order to characterize RVM cells. A trial was not initiated if an ON or OFF cell showed constant background activity for at least 10 s. Once an RVM cell had been identified as ON, OFF or neutral, we optimised spike size before any treatment. This study only included neurons whose spike configuration remained constant and could clearly be discriminated from activity in the background throughout the experiment, indicating that the activity from a single neuron was measured. However this study did not include recordings of the neutral cells that were encountered during the cell search (n = 24 in healthy, n = 8 in sham and n = 8 in SNI rats, corresponding to 16.2, 15.8 and 14.26% of the cells encountered in healthy, sham and SNI groups, respectively). The recording site was marked with a 20 μA DC current for 20 s. After fixation by immersion in 10% formalin, the recording sites were identified. In each rat, only one neuron was recorded before and after vehicle or drug administration. Neuron responses both before and after intra-VL PAG vehicle or drug microinjections were measured and expressed as spikes/sec (Hz). At the end of the experiment, each animal was killed with a lethal dose of urethane, the microinjection site was marked with 0.2 μl of a Cresyl Violet solution and the recording site marked with a 20 μA DC current for 20 s. After fixation by immersion in 10% formalin, the microinjection and recording sites were identified.

### Treatments

Healthy animals receiving a single intra-VL PAG administration of 200 nl vehicle (DMSO/ACSF, 0.05%, v/v), ONO-DI-004 (Ki = 0.15 μM and EC_50 _= 0.42 μM at EP1 receptor, [22]), PGE2 or L335677 (Ki = 15 nM with a selectivity of at least 100-fold against EP2, EP4, FP and IP and 67-, 11-, and 10-fold against EP3, DP and TP receptors, respectively [23]) were grouped as follows:

a) Group 1 rats receiving vehicle;

b) Group 2 rats receiving different doses of ONO-DI-004 (0.001, 0.01 and 0.1 pg), a selective EP1 receptor agonist;

c) Group 3 rats receiving 0.1 pg of ONO- DI-004 in combination with L-335677 (0.1 pg), a selective EP1 receptor antagonist;

d) Group 4 rats receiving different doses of PGE2 (0.01, 0.1 and 1 pg);

e) Group 5 rats receiving 1 pg of PGE2 in combination with L-335677 (0.1 pg);

f) Group 6 rats receiving different doses of L-335677 (0. 1, 1 and 10 pg);

g) Group 7 rats receiving ONO-DI-004 (0.1 pg) intentionally outside the VL PAG.

Sham and SNI animals receiving a single intra-VL PAG administration of 200 nl vehicle (DMSO/ACSF, 0.05%, v/v) or drug solutions 7 days after surgery were grouped as follows:

1) Sham and SNI rats receiving vehicle;

2) Sham and SNI rats receiving ONO-DI-004 (0.1 pg);

3) Sham and SNI rats receiving L-335677 (10 pg).

Since only one neuron was recorded in each rat, experimental groups consisted of 12-14 rats in order to record at least 6-7 ON and 6-7 OFF cells for each treatment. Only group *g *consisted of 7 rats which were used for monitoring tail flick latency only, in order to verify the specificity of the effect of the microinjection site. When L-335677 was used in combination with ONO-DI-004 or PGE2, the latters were administered 10 minutes after L-335677.

### Statistics

Behavioural data were expressed as means ± S.E.M of latency time to the tail withdrawal reflex. RVM background cell activity was expressed as means ± SEM of the spikes/s obtained by averaging the ongoing cell firing recorded in the 50 s prior to tail flick trials (which were carried out every 5 min). ON cells identified by a burst of activity just before tail flick responses were spontaneously active in 31.9% of cases and inactive in the remaining cases [24]. ON cells with spontaneous activity were the only cells investigated and included in the data analysis to characterize the activity of this ON cell subgroup more accurately and to consider post drug changes in their spontaneous activity. Tail flick-related ON cell burst was calculated as means ± S.E.M. of the number of spikes in the 10 sec interval starting from the onset of the increase in cell frequency (which was at least double its spontaneous activity). Furthermore, the onset of the ON cell burst was calculated as means ± S.E.M. (s) of time elapsing between the application of the noxious radiant heat and the beginning of the tail flick-related increase in cell frequency. The onset of the OFF cell pause was calculated as means ± S.E.M of the time (s) elapsing between the onset of the application of thermal stimulus and the last spike. Finally, the duration of the cell pause was expressed as means ± S.E.M. of the time (s) elapsing between the pause onset and the first spike after the tail flick. For tail flick latency and electrophysiology data, comparisons were made using 2-way ANOVA for repeated measures, followed by the Newman-Keuls *post-hoc *test. ANOVA followed by the Tukey *post-hoc *test were used for immunohistochemistry and western blot analysis. P < 0.05 was considered statistically significant.

### Drugs

ONO-DI-004 was kindly provided by ONO Pharmaceutical Co Ltd, Osaka, Japan. (5Z, 11α, 13E, 15S)-11, 15-dihydroxy-9-oxo-prosta-5, 13-dien1oic acid (Prostaglandin E2) was purchased from Tocris Bioscience, Bristol, UK. (3-{3-[2-(benzyloxy)-5-chlorophenyl]-2-thienyl}phenyl) acetic acid (L-335677) was kindly provided by Merck Frosst Canada and Co., Quebec, Canada. All drugs were dissolved in 0.05% DMSO in ACSF.

## Results

### EP1 expression and localization in the VL PAG

The immunohistochemical approach was used to reveal EP1 expression and localization within the VL PAG (Figure 1A). Western blot analysis demonstrated the presence of EP1 receptor within the VL PAG (Figure 1B). Western blot analysis showed a significant decrease in EP1 receptor protein levels in the VL PAG of SNI rats (10004.25 ± 309.42, n = 6, p < 0.05, one-way ANOVA/Tukey test) compared with the shams (mean of arbitrary units ± SEM: 21375.5 ± 1041.5, n = 6) 7 days after sciatic nerve surgery (Figure 1B'). Double labelling revealed that EP1 receptors were mostly expressed in the VGAT labelled neurons, while few EP1 positive profiles were present in the VGluT1 labelled in the VL PAG (Figure 1C).

### Effect of intra-VL PAG ONO-DI-004 and L335677 on tail flick latencies

**Figure 2:**
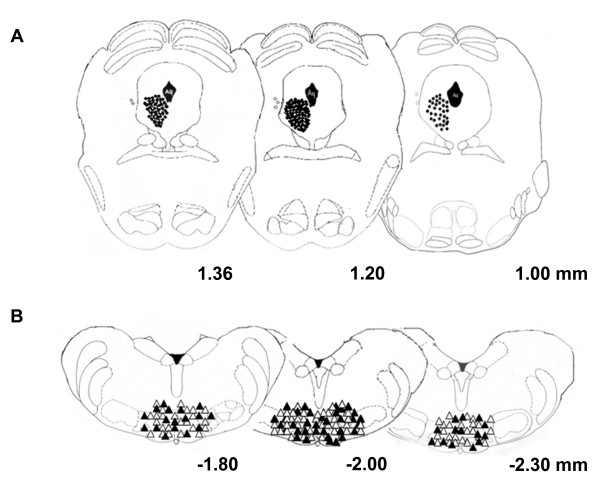
**Histologically verified microinjection sites for drug or vehicle microinjections in the VL PAG (A) and recording sites for ON and OFF cells in the RVM (B), in transverse sections simplified by Paxinos and Watson **[20]. Vehicle or drug microinjections are depicted with filled circles (A). The open circles indicate microinjections that were accidentally or intentionally performed outside of VL PAG, the effects of which (n = 7) were considered in the study for location specificity. Triangles indicate electrode tips in the RVM (B); in particular white triangles represent ON cells and the black ones represent the OFF cells. Distances (in mm) from the interaural line are indicated.

Only rats whose microinjected site was located within the VL PAG (black circles) were used for data computation (Figure 2A). Cannulae were also intentionally implanted 1 mm outside from VL PAG for microinjection site controls (white circles).

**Figure 3:**
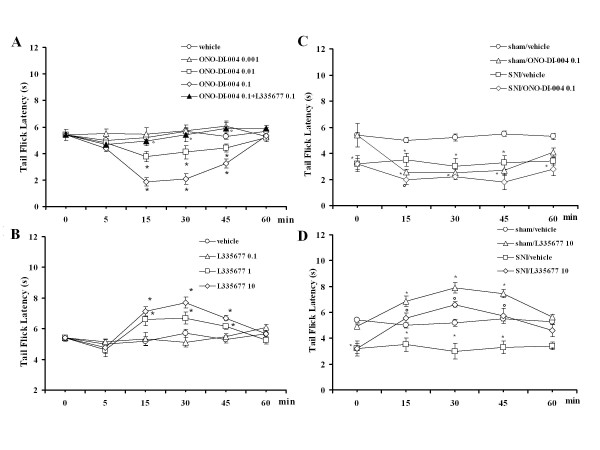
**Tail flick latencies after intra-VL PAG microinjections of vehicle (0.05% DMSO in ACSF), ONO-DI-004 (0.001, 0.01 and 0.1 pg) alone, or ONO-DI-004 (0.1 pg) in combination with L355677 (0.1 pg) (A) or L335677 (0.1, 1 and 10 pg) (B)**. Tail flick latencies were also measured in sham and SNI rats. In particular, *C *shows the effect of intra-VL PAG microinjections of vehicle (0.05% DMSO in ACSF) or ONO-DI-004 (0.1 pg) and *D *indicates the effects of vehicle or L335677 (10 pg) in sham and SNI rats **7 days after SNI surgery**. Each point represents the mean ± S.E.M of 12-14 rats per group. * indicates a statistically significant difference vs vehicle and° vs ONO-DI-004 (0.1 pg) in *A *and *B*. * indicates a statistically significant difference vs sham/vehicle and° vs SNI/vehicle in *C *and *D*. P values < 0.05 were considered statistically significant.

Tail flicks were elicited every 5 min for 15 min prior to microinjecting drugs or respective vehicle into the VL PAG. Data related to pre-treatment intervals were considered as basal tail flick latencies (5.5 ± 0.6 s). Intra-VL PAG microinjections of vehicle did not change tail flick latency (5.2 ± 0.7 s, n = 12) compared with basal values. Tail flick latency was significantly reduced to 3.7 ± 0.4 s and 1.86 ± 0.2 s (*P *< 0.05; F(3-51) = 27.01; n = 14 for each dose) by intra-VL PAG microinjections of ONO-DI-004 (0.01 and 0.1 pg) respectively, while the lowest dose (0.001 pg) did not significantly change (n = 12) the nocifensive response (Figure 3A). The pronociceptive effect induced by the highest dose of ONO-DI-004 (0.1 pg) was completely prevented (n = 12) by 10 min pre-treatment with L335677 (0.1 pg) (Figure 3A), which was inactive per se at this dose (n = 13) (Figure 3B). Intra-PAG administrations of L335677 (1 and 10 pg) significantly increased the tail flick latency to 6.7 ± 0.4 and 8 ± 0.35 s (*P *< 0.05; F(3-41) = 14.06; n = 12 for both doses)(Figure 3B). ONO-DI-004 (0.1 pg) was also intentionally microinjected 1 mm from the PAG (n = 7), where it failed to change tail flick latency (not shown).

### Effect of intra-VL PAG ONO-DI-004 and L335677 on tail flick latencies in sham and SNI rats

In sham and SNI rats, basal tail flick latencies were similar to pre-treatment intervals (5.3 ± 0.8 s, n = 7 and 3.1 ± 0.6 s, n = 13 respectively). Intra-VL PAG microinjection of vehicle did not change tail flick latency in sham or SNI rats (5.2 ± 0.15 s, n = 12 and 3.2 ± 0.7 s, n = 12, respectively) compared to basal values. Intra-VL PAG microinjection of ONO-DI-004 (0.1 pg) significantly reduced the tail flick latency to 2.7 ± 0.28 s (*P *< 0.05; F(1-23) = 55.71; n = 13) and 2 ± 0.2 s (*P *< 0.05; F(1-24) = 10.9; n = 12) in sham and SNI rats respectively (Figure 3C). Tail flick latency was significantly increased to 7.9 ± 0.4 s (*P *< 0.05; F(1-19) = 42.99; n = 12) and 6.6 ± 0.8 s (*P *< 0.05; F(1-22) = 12.96; n = 12) by intra-VL PAG microinjections of L-335677 (10 pg) in sham and SNI rats respectively (Figure 3D).

### Effect of intra-VL PAG PGE2 on tail flick latencies

**Figure 4:**
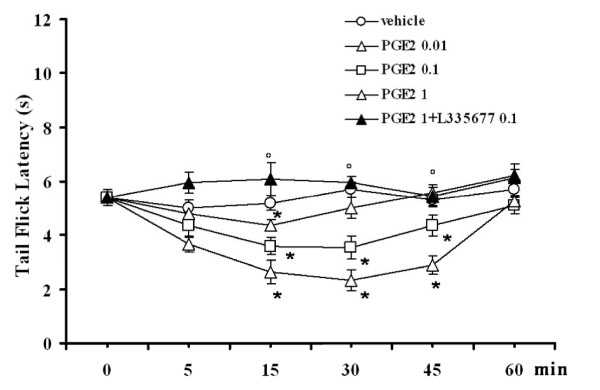
**Tail flick latencies after intra-VL PAG microinjections of vehicle (0.05% DMSO in ACSF), PGE2 (0.01, 0.1 and 1 pg) or PGE2 (1 pg) in combination with L355677 (0.1 pg)**. Each point represents the mean ± S.E.M of 12-14 rats per group. * indicates a statistically significant difference vs vehicle and° versus PGE2 (1 pg). P values < 0.05 were considered statistically significant.

Tail flick latency was significantly reduced to 3.5 ± 0.3 s and 2.3 ± 0.4 s (*P *< 0.05; F(4-65) = 14.85; n = 14 for both doses) by microinjection of PGE2 (0.1 and 1 pg), respectively. The lowest dose of PGE2 (0.01 pg) only significantly changed the nocifensive response at 15 min post injection (n = 12). The pronociceptive effect induced by the highest dose of PGE2 (1 pg) was completely prevented by 10 min pre-treatment with L-335677 (0.1 pg) (n = 13), which was inactive per se (Figure 4).

### Effect of ONO-DI-004 and L-335677 on the ongoing activity of RVM ON and OFF cells

**Figure 5:**
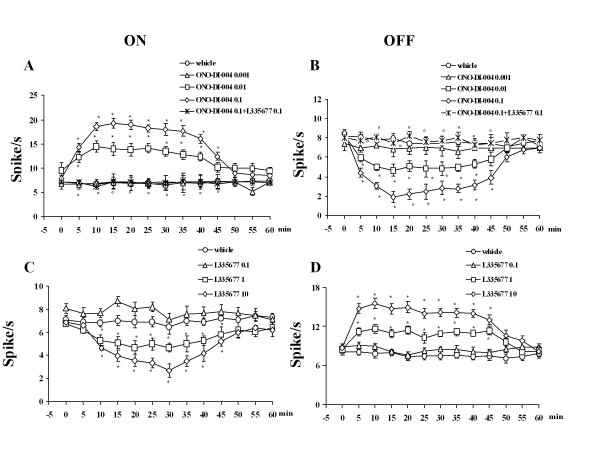
**The effects of intra-VL PAG microinjections of vehicle (0.05% DMSO in ACSF), ONO-DI-004 (0.001, 0.01 and 0.1 pg) alone, or ONO-DI-004 (0.1 pg) in combination with L355677 (0.1 pg) (A and B) or L355677 (0.1, 1 and 10 pg) (C and D), on the spontaneous firing of RVM ON (A and C) or OFF (B and D) cells**. Vehicle or drugs were administered at time 0, whereas L 335677 (0.1 pg) was administered 10 min beforehand (A and B). Each point represents the mean ± S.E.M of 6-7 neurons. * indicates a statistically significant difference vs vehicle and° versus ONO-DI-004 (0.1 pg). P values < 0.05 were considered statistically significant.

The results are based on RVM neurons (one cell recorded from each animal per treatment) at a depth of 9, 900-10, 955 μm from the surface of the brain. All recorded neurons identified as ON cells by a burst of activity immediately prior to tail flick responses were spontaneously active in 29.2% of cases and inactive in the remaining ones. Only ON cells showing spontaneous activity were chosen and included in the analysis of the data so as to characterize the activity of this ON cell subgroup more accurately and to consider post-drug changes in their spontaneous activity. This population of ON cells had a mean frequency of spontaneous activity of 7.3 ± 0.5 spikes/s (Figure 5A). ON cells with tail flick-evoked activity only showed a mean spontaneous activity of 0.38 ± 0.3 spikes/s and were not included in data analysis. OFF cells showed a mean spontaneous activity of 8.1 ± 0.7 spikes/s. Microinjection of vehicle (0.05% DMSO in ACSF) did not change the spontaneous activity of the ON (7.6 ± 0.3 spikes/s, n = 6) (Figure 5A) or OFF cells (8.1 ± 0.7 spikes/s, n = 6) (Figure 5B). Intra-VL PAG microinjections of ONO-DI-004 (0.01 and 0.1 pg) caused an increase in ON cell activity, which was significant between 5 and 40 min (14.4 ± 0.8 spikes/s) and 5 and 45 min (19.2 ± 0.7 spikes/s), (*P *< 0.05, F(3-25) = 40.00; n = 7 for both doses) respectively (Figure 5A). ONO-DI-004 (0.01 and 0.1 pg) decreased OFF cell activity and this effect was significant between 5 and 45 min in both cases (5.0 ± 0.4 and 2.2 ± 0.6 spikes/s, respectively) (P < 0.05; F(3-25) = 17.54; n = 7 for both doses) (Figure 5B). The lowest dose of ONO DI-004 (0.001 pg) did not change the spontaneous activity of ON or OFF cells (n = 6 for both cells). The effects of ONO-DI-004 (0.1 pg) on the ongoing activity of ON and OFF cells were completely abolished by 10 min pre-treatment with L335677 (0.1 pg, n = 6 for both cells) (Figure 5A and 5B).

Intra-VL PAG microinjections of L-335677 (1 and 10 pg) caused a decrease in the spontaneous firing activity of the ON cells, which was already significant after 10 min and lasted for 45 min at both doses (4.6 ± 0.4 and 2.7 ± 0.6 spikes/s, respectively) (P < 0.05; F(3-23) = 22.90; n = 6 for both doses) (Figure 5C). L-335677 (1 and 10 pg) also increased the spontaneous activity of OFF cells, which was significant between 5 and 45 min at both doses (11.6 ± 0.6 and 15.4 ± 0.8 spikes/s, respectively) (P < 0.05; F(3-23) = 31.58; n = 6 for both doses) (Figure 5D). The lowest dose of L335677 (0.1 pg) did not alter the spontaneous activity of either ON (n = 7) or OFF (n = 6) cells (Figure 5C and 5D).

### Effect of ONO-DI-004 and L-335677 on the ongoing activity of RVM ON and OFF cells in sham and SNI rats

In sham and SNI rats, the ON cell population showed a mean frequency of spontaneous activity of 7.2 ± 0.5 and 14.8 ± 1.5 spikes/s, respectively. Therefore the mean frequency of ON cells in the sham rats did not differ from that of healthy rats (7.3 ± 0.5 spikes/s), whereas the mean frequency of ON cell activity in SNI rats significantly increased compared to both sham and healthy rats (P < 0.05; F(1-16) = 27.66; n = 6 and P < 0.05; F(1-20) = 25.43; n = 7, respectively). Microinjection of vehicle (0.05% DMSO in ACSF) did not change the spontaneous activity of ON cells (7.1 ± 0.8 spikes/s, n = 6 and 15 ± 1.3 spikes/s, n = 7) in sham or SNI rats, respectively. The intra-VL PAG microinjection of ONO-DI-004 (0.1 pg) caused an increase in the spontaneous firing activity of ON cells, which was significant after 5 min and lasted 45 and 60 min (19.2 ± 1.4 spikes/s, P < 0.05; F(1-15) = 52.56; n = 6 and 22.5 ± 2.8 spikes/s, P < 0.05; F(1-19) = 5.52; n = 7) in sham and SNI rats, respectively. The population of OFF cells had a mean frequency of spontaneous activity of 8 ± 0.3 and 4.8 ± 0.5 spikes/s in sham and SNI rats, respectively. The mean frequency of the OFF cells in the sham rats therefore did not differ from that of healthy rats (8.1 ± 0.7 spikes/s), whereas the mean frequency of OFF cell activity in SNI rats was significantly reduced (4.4 ± 0.7 spikes/s), compared to both sham and healthy rats (*P *< 0.05; F(1-22) = 6.92; n = 6 and *P *< 0.05; F(1-22) = 4.66; n = 7, respectively). The microinjection of vehicle (0.05% DMSO in ACSF) did not change the spontaneous activity of OFF cells (8.1 ± 0.9 spikes/s, n = 6; 3.7 ± 0.5 spikes/s, n = 7) in sham and SNI rats, respectively. Intra-VL PAG microinjections of ONO-DI-004 (0.1 pg) caused a decrease in the spontaneous firing activity of OFF cells that was already significant between 5 min and 50 min and between 10 and 60 min (3 ± 0.5 spike/s, P < 0.05; F(1-19) = 24.54; n = 6 and 2.2 ± 0.4 spikes/s, P < 0.05; F(1-21) = 22.69; n = 7) in sham and SNI rats, respectively.

Intra-PAG microinjections of L335677 (10 pg) caused a reduction in ON cell activity that was significant between 10 and 45 min and 5 and 60 min (3.9 ± 0.6 spikes/s, P < 0.05; F(1-22) = 10.67; n = 7 and 5.2 ± 0.8 spikes/s, P < 0.05; F(1-19) = 39.25; n = 7) in sham and SNI rats, respectively. Intra-PAG microinjections of L335677 (10 pg) caused an increase in OFF cell activity which was significant between 10 and 50 min and 5 and 60 min (15.5 ± 0.83 spikes/s, *P *< 0.05; F(1-20) = 35.73; n = 7; 11.7 ± 1.1 spikes/s, *P *< 0.05; F(1-18) = 33.75, n = 7) in sham and SNI rats, respectively.

### Effect of PGE2 on the ongoing activity of RVM ON and OFF cells

**Figure 6:**
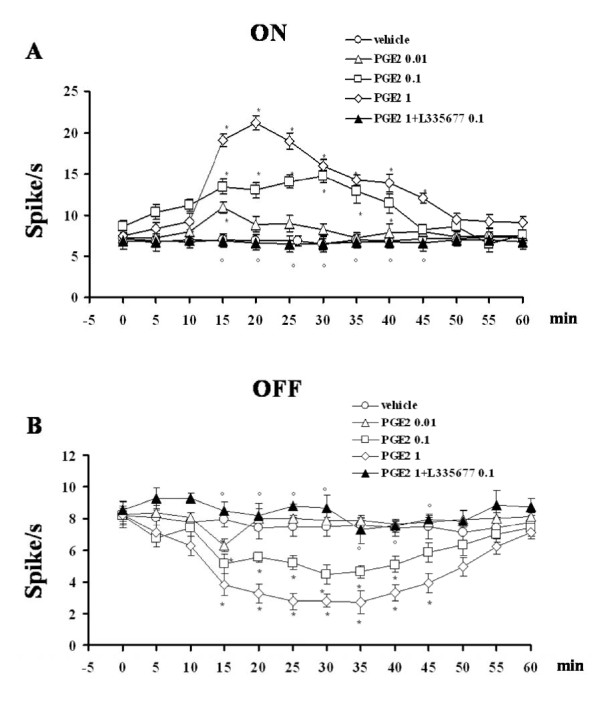
**Effects of intra-VL PAG microinjections of vehicle (0.05% DMSO in ACSF), PGE2 (0.01, 0.1 and 1 pg) alone, or PGE2 (1 pg) in combination with L355677 (0.1 pg) on the spontaneous firing of RVM ON (A) or OFF (B) cells**. Vehicle or drugs were administered at time 0, whereas L 335677 (0.1 pg) was administered 10 min beforehand. Each point represents the mean ± S.E.M of 6-7 neurons of different treated groups of rats. * indicates a statistically significant difference vs vehicle and° versus PGE2 (1 pg). P values < 0.05 were considered statistically significant.

Intra-PAG microinjections of PGE2 (0.01, 0.1 and 1 pg) caused an increase in ON cell activity that was significant at 15 min (10.9 ± 0.7 spikes/s, n = 6), between 15 and 40 min (14.7 ± 0.8 spikes/s) and between 15 and 45 min (21.2 ± 1.3 spikes/s), respectively (P < 0.05; F(3-23) = 42.20; n = 7 for the higher doses) (Figure 6A). Similar treatment reduced OFF cell activity, with a significant effect at 15 min (6.3 ± 0.4 spikes/s, n = 6), between 15 and 40 (3.9 ± 0.5 spikes/s) and between 15 and 45 min (1.4 ± 0.5 spikes/s), respectively (P < 0.05; F(3-23) = 64.66; n = 7 for the higher doses) (Figure 6B). The effects of PGE2 (1 pg) on ongoing ON (n = 6) and OFF cell (n = 7) activity were completely abolished by 10 min pre-treatment with L335677 (0.1 pg) (Figure 6A and 6B).

### Effect of intra-VL PAG ONO-DI-004 and L-335677 on tail flick-related ON and OFF cell activity

**Figure 7:**
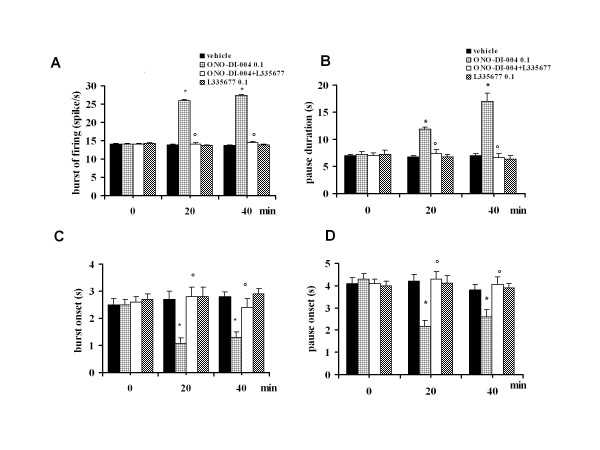
**The effects of intra-VL PAG microinjections of vehicle (0.05% DMSO in ACSF), ONO-DI-004 (0.1 pg) alone, or in combination with L355677 (0.1 pg) on the ON cell tail flick-evoked burst of firing (A) and onset of the burst (C) or OFF cell tail flick-evoked pause (B) and onset of the pause (D)**. Each histogram represents the mean ± S.E.M of 6-7 neurons of different treated groups of rats. * indicates significant differences vs vehicle and° versus ONO-DI-004 (0.1 pg). P values < 0.05 were considered statistically significant.

Microinjections of vehicle did not change the tail flick-induced ON cell burst (14.08 ± 1.8 spikes/s, n = 6), OFF cell pause (6.95 ± 2.4 s, n = 6) (Figure 7A and 7B) or the onset of the burst (2.5 ± 1.4 s) and pause (4.1 ± 2.1 s) (Figure 7C and 7D). Intra-VL PAG microinjections of ONO-DI-004 (0.01 and 0.1 pg) caused an increase in both the ON cell burst (22 ± 1.3 spikes/s and 27.4 ± 0.9 spikes/s, respectively) (P < 0.05; F(2-20) = 8.81; n = 7 for both doses) and OFF cell pause (12.8 ± 0.4 s and 17 ± 1.5 s) (F(2-19) = 25.32; P < 0.05; n = 7 for both doses) (Figure 7A and 7B for the highest dose only). Furthermore, ONO-DI-004 (0.01 and 0.1 pg) caused a decrease in the onset of burst to 1.7 ± 0.3 s and 1.3 ± 0.2 s, respectively (P < 0.05; F(2-19) = 7.98; n = 7 for both doses) and in the onset of the OFF cell pause to 3.4 ± 0.2 s and 2.6 ± 0.3 s, respectively (P < 0.05; F(2-19) = 5.83; n = 7 for both doses) (Figure 7C and 7D). The effects induced by the highest dose of ONO-DI-004 (0.1 pg) were completely prevented by pre-treatment with the lowest dose of L-335677 (0.1 pg) (n = 6 for both cells), which was inactive per se (n = 7 for the ON cells and n = 6 for OFF cells) (Figure 7A and 7B). ONO-DI-004 (0.001 pg) did not alter tail flick-evoked ON and OFF cell activity (n = 6 for both doses) (not shown).

### Effect of intra-VL PAG ONO-DI-004 and L-335677 on tail flick-related ON and OFF cell activity in sham and SNI rats

**Figure 8:**
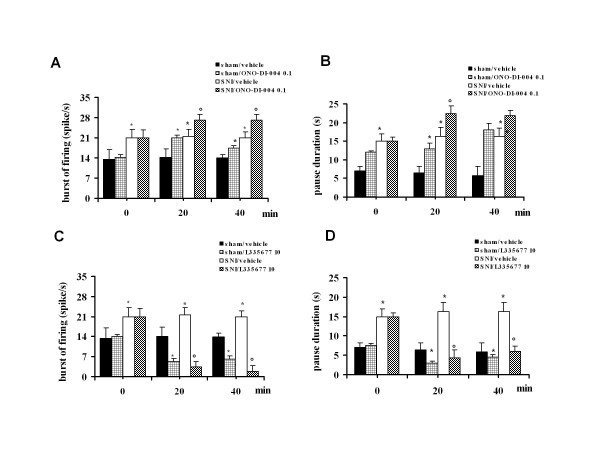
**The effects of intra-VL PAG microinjections of vehicle (0.05% DMSO in ACSF), ONO-DI-004 (0.1 pg) (A, C) or L355677 (10 pg) (B, D) on the ON cell tail flick-evoked burst of firing (A and B) and OFF cell tail flick-evoked pause (C and D) in sham and SNI rats 7 days after surgery**. Each histogram represents the mean ± S.E.M of 6-7 neurons of different treated groups of rats. * indicates significant differences vs sham/vehicle and° versus SNI/vehicle. P values < 0.05 were considered statistically significant.

In sham and SNI rats, the population of ON cells had a tail flick-induced burst of firing of 14.1 ± 1.2 and 22 ± 2.8 spikes/s, respectively (Figure 8A and 8B). Therefore the mean of the frequency of the ON cell burst in the sham rats did not differ from that of healthy rats (14.08 ± 1.8 spikes/s, n = 6) whereas the mean frequency of the ON cell burst in SNI rats was significantly increased compared to both sham and healthy rats (P < 0.05; F(1-14) = 18.77; n = 6 and P < 0.05; F(1-13) = 18.77; n = 7, respectively). The population of OFF cells showed a pause of 6.3 ± 1.3 s and 15.8 ± 2.5 s in sham and SNI rats, respectively (Figure 8C and 8D). The duration of the OFF cell pause in the sham rats did not therefore differ from that of healthy rats (6.65 ± 2.45 s), whereas the pause of OFF cells in SNI rats significantly increased compared to both sham and healthy rats (*P *< 0.05; F(1-16) = 6.26; n = 6 and *P *< 0.05; F(1-15) = 10.28; n = 7, respectively). Microinjections of vehicle in sham and SNI rats did not change the tail flick-induced ON cell burst (14.0 ± 1.2, n = 6 and 21.8 ± 1.1 spikes/s, n = 6, respectively) (Figure 8A and 8B) or OFF cell pause (5.9 ± 1.4 s, n = 6, and 15.6 ± 2.2 s, n = 6, respectively) (Figure 8C and 8D). Intra-VL PAG microinjections of ONO-DI-004 (0.1 pg) caused an increase in both the ON cell burst (23.1 ± 0.9 spikes/s, P < 0.05; F(1-10) = 36.8; n = 7 and 26 ± 0.37 spikes/s, P < 0.05; F(1-10) = 14.5; n = 6, respectively) and OFF cell pause (18.5 ± 1.8 s, P < 0.05; F(1-11) = 26.70; n = 7 and 22 ± 1.55 s, P < 0.05; F(1-21) = 5.8; n = 7, respectively) (Figure 8A and 8C) in sham and SNI rats, respectively. Intra-VL PAG microinjections of ONO-DI-004 (0.1 pg) caused a decrease in the onset of the ON cell burst (1.3 ± 0.1 s and 0.22 ± 0.06 s, P < 0.05; F(1-12) = 54.44; n = 6 and P < 0.05; F(1-12) = 4.41; n = 6) in sham and SNI rats, respectively. Intra-VL PAG microinjections of ONO-DI-004 (0.1 pg) also caused a decreasing of the onset of pause to 2.6 ± 0.3 s and 0.2 ± 0.09 s (P < 0.05; F(1-12) = 11.68; n = 6 and P < 0.05; F(1-12) = 5.62; n = 6 (Figure 8D) in sham and SNI rats, respectively.

**Figure 9:**
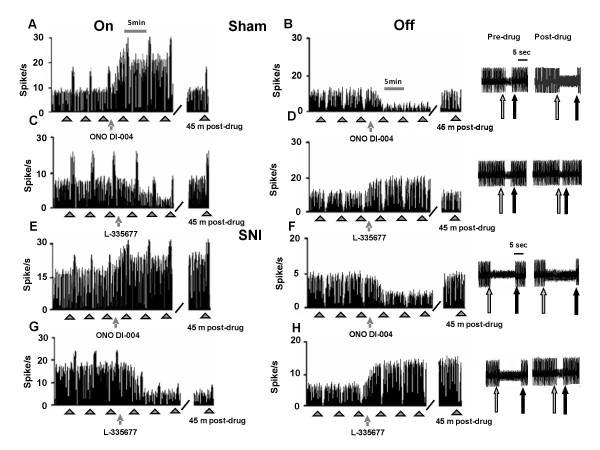
**Examples of ratemater records which illustrate the effect of intra-VL PAG microinjection of ONO-DI-004 (0.1 pg) or L335677 (10 pg) on either the ongoing or tail flick-related burst of activity of identified RVM ON cells (A, C, E and G) and ongoing or tail flick-related pause of identified RVM OFF cells (B, D, F and H) in sham (A, B, C and D) and SNI (E, F, G and H) rats**. Intra-VL PAG microinjection of ONO-DI-004 (0.1 pg) increased the ongoing activity and tail flick-related burst of the ON cells in sham and SNI rats (A and E, respectively). The same treatment reduced the ongoing activity and increased the tail flick-related pause of the OFF cells in sham and SNI rats (B and F, respectively). Conversely, intra-VL PAG microinjection of L335677 (10 pg) reduced the ongoing activity and tail flick-related burst of the ON cells both in sham and SNI rats (C and G, respectively). The same treatment increased the ongoing activity and reduced the tail flick-related pause of the OFF cells in both sham and SNI rats (D and H, respectively). Scales bars indicate 5 min for ratemater records, while small full arrows indicate the noxious stimulation. At the right a time expanded scale illustrates pause duration changes (scale bar = 5 sec). The grey arrows show the noxious stimuli application and the black one the tail flick reflex.

Intra-VL PAG microinjections of L-335677 (10 pg) reduced the burst of firing (5.3 ± 1 spike/s and 3.2 ± 1.28 spike/s) (P < 0.05; F(1-10) = 37.85; n = 6 and P < 0.05; F(1-10) = 45.48 n = 6) in sham and SNI rats respectively (Figure 8B) and increased the onset of ON cell burst (4 ± 1 s and 0.76 ± 0.04 s) (P < 0.05; F(1-12) = 114.89; n = 6 and P < 0.05; F(1-12) = 13.61; n = 6) in sham and SNI rats, respectively. Intra-VL PAG microinjections of L-335677 (10 pg) also reduced the OFF cell pause to 2.9 ± 0.6 s and 4.3 ± 1.3 s (P < 0.05; F(1-12) = 7.4; n = 6 and P < 0.05; F(1-12) = 19.5, n = 6)in sham and SNI rats, respectively (Figure 8D) as well as increasing the onset of pause to 6.7 ± 0.4 s and 0.88 ± 0.08 s (P < 0.05; F(1-12) = 183.2; n = 6 and P < 0.05; F(1-12) = 12.35; n = 6 (Figure 8D) in sham and SNI rats, respectively. Representative ratemeter records showing tail flick-related activity of ON and OFF cells in sham and CCI rats before and after ONO-DI-004 (0.1 pg) or L335677 (10 pg) are shown in Figure 9.

### Effect of intra-PAG PGE2 on tail flick-related ON and OFF cell activity

**Figure 10:**
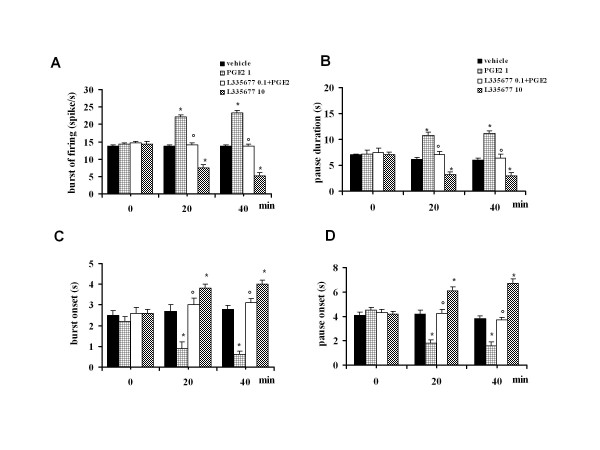
**The effects of intra-VL PAG microinjections of vehicle (0.05% DMSO in ACSF), L335677 (10 pg), PGE2 (1 pg) alone, or PGE2 (1 pg) in combination with L355677 (0.1 pg) on the ON cell tail flick-evoked burst of firing (A) and onset of the burst (C), or OFF cell tail flick-evoked pause (B) and onset of the pause (D)**. Each histogram represents the mean ± S.E.M of 6-7 neurons of different treated groups of rats. * indicates significant differences vs vehicle and° versus PGE2 (1 pg). P values < 0.05 were considered statistically significant.

Intra-VL-PAG microinjections of PGE2 (0.01, 0.1 and 1 pg) caused an increase in the ON cell burst (15.8 ± 1 spike/s, n = 6, 19.2 ± 1.1 spike/s, n = 7 and 23.4 ± 1.2 spikes/s, n = 7, respectively) (P < 0.05; F(3-25) = 17.36) (Figure 10A for the highest dose only) and OFF cell pause (6.8 ± 0.8 s, n = 6, 9.9 ± 0.7 s, n = 7 and 11.2 ± 0.4 s, n = 7, respectively) (P < 0.05; F(3-22) = 18.31) (Figure 10B for the highest dose only). PGE2 (0.01, 0.1 and 1 pg) also caused a reduction in the onset of burst to 2.5 ± 0.2 s (n = 6), 1.8 ± 0.3 s (n = 7) and 0.6 ± 0.2 s (n = 7) (P < 0.05; F(3-25) = 18.16), respectively (Figure 10C for the highest dose only) and in the OFF cell pause to 4.4 ± 0.2 s (n = 6), 2.8 ± 0.3 s (n = 7) and 1.5 ± 0.3 s (n = 7), respectively (P < 0.05; F(3-25) = 17.36) (Figure 10D for the highest dose only). The effects induced by the highest dose of PGE2 (1 pg) were completely prevented by pre-treatment with the lowest dose of L-335677 (0.1 pg, n = 6 for the ON and n = 7 for the OFF cells), which was inactive per se (n = 7 for the ON and n = 6 for the OFF cells) (Figure 10).

## Discussion

It is widely agreed that prostaglandins contribute to nociception facilitation through sensitization of primary afferent sensory neurons at the site of injury and spinal circuitry [3,25-27]. The role of supraspinal PGE2 in pain facilitation remains less explored despite studies which have demonstrated the presence of PGE2 receptors in the hypothalamus, hippocampus and PAG [6,7]. A particularly strong positivity to the polyclonal antibody against an amino-terminal portion of EP3 receptor and mRNA have been found within the PAG [7]. The presence of EP1 receptor, whose role in pain facilitation has previously been established [12-17], has instead never been ascertained within the PAG. Previous evidence has suggested a facilitatory role of PGE2 within the PAG on pain transmission [10,11]. One of our previous studies has shown that VL PAG EP1 receptor blockade inhibited pain responses or misoprostol-induced effect [12]. In the present study, the expression of EP1 receptor in the VL PAG was ascertained by western blot and immunohistochemistry. Our results demonstrate that EP1 receptors were widely expressed within the VL PAG and co-localize with the vesicular GABA transporter (VGAT) positive profile. The GABAergic neural population constitutes ~ 50% of total neural elements (the majority are active tonic interneurons) of the PAG controlling its intrinsic activity which affects the antinociceptive descending circuitry function [28,29]. EP1 receptor is associated with Ca^2+ ^mobilization and neurotransmitter release [3,18]. Consistent with a previous finding in the striatum suggesting that EP1 receptor can be expressed on either the presynaptic or postsynaptic membrane [30], EP1 receptor stimulation in our study could be associated with GABA release. An increase in GABA release within the VL PAG has already been shown for misoprostol, a non-specific prostaglandin analogue [12], which activates all EP receptors. In another study, sulprostone, an EP3 receptor selective agonist, and PGE2 inhibited miniature excitatory postsynaptic currents (mEPSCs), with no action on inhibitory postsynaptic currents (mIPSCs), demonstrating the role of EP3 receptor in decreasing glutamate release from presynaptic sites [31]. Changes in excitatory and/or inhibitory neurotransmitters have a relevant meaning within the PAG, since its stimulation and/or depression is consistent with pain inhibition and/or facilitation. Moreover, despite the potential for persistent exposure to prostaglandins **at **the peripheral site of injury or within the CNS, few studies have been performed to assess the effect of prostaglandin receptor expression and mediated responses in chronic pain conditions [32]. In neuropathic pain conditions induced by the SNI of the sciatic nerve, a reduced expression of EP1 receptor was observed within the VL PAG 7 days after the surgery. A significant decrease of EP receptor in chronic pain conditions has already been observed in the DRG, spinal cord dorsal horn and sensory neurons [32,33]. EP receptor downregulation appeared to be PGE2-mediated, since it was abolished by intrathecal administration of ketoralac, an inhibitor of the COXs, which proved to be overexpressed in inflammatory pain conditions [34,35].

Selective stimulation of EP1 receptor by intra-VL PAG microinjection of ONO-DI-004, a selective EP1 receptor agonist, as well as the stimulation of all EP receptors by PGE2, decreased the latency of the thermoceptive reaction. The facilitation of nociceptive responses by intra-VL PAG PGE2 is consistent with previous studies demonstrating that the direct application of PGE2 within the VL PAG was able to facilitate nociceptive responses in lightly anaesthetized rats [10,11]. Here, as well as confirming this previous evidence using a different themoceptive test; the tail flick, we also show that selective EP1 stimulation by ONO-DI-004 is able to induce the facilitation of nociception. Moreover, this study shows that L335677, a selective EP1 receptor antagonist, which was unable to alter the thermal threshold at a low dose, completely prevented the effects of intra-VL PAG microinjections of ONO-DI-004 and PGE2. The fact that EP1 selective antagonist completely prevents the PGE2-induced effect suggests that the PGE2-induced hyperalgesic effect requires the involvement of EP1 receptor. This dependence of large spectrum EP receptor agonist on each EP receptor has already been emerged with misoprostol, a synthetic PGE2 analogue, whose effect has been shown to require EP1, EP2, EP3 and EP4 receptor stimulation [12].

Moreover EP1 receptor blockade by L335677 (at higher doses than those used for antagonizing ONO-DI-004 and PGE2 effects) increased per se the latency of the thermoceptive reaction. Since EP1 receptor blockade by L335677 produced antinociception, it appears that this receptor is under tonic activation in facilitating the nociceptive response within the VL PAG. Therefore taken together, this study and previous ones [10,11] suggest that apart from performing an inhibitory role, VL PAG also mediates facilitatory pain control. A tonic facilitatory role of EP1, EP2, EP3 and EP4 receptors, whose blockade determined analgesia, has already been found in formalin-induced persistent pain conditions [12]. However, it has scarcely been investigated in neuropathic pain conditions [17]. ONO-DI-004 and L335667 were both effective in changing the nociceptive reaction in a facilitatory and inhibitory manner, respectively in neuropathic pain conditions in this study. In this study it is significant to observe that neuropathic pain, which leads to almost a 50% reduction in EP1 receptor expression, did not alter the effect of EP1 ligands in changing pain response in the VL PAG according to a previous study in the sensory neurons and spinal cord [32]. Possible explanations for the maintained pain-related activity of EP1 receptor stimulation/blockade in the face of its downregulation may be due to: i) the presence of spare receptors whose reduction in expression does not affect their related response; ii) loss of receptor on a certain cell population not involved in controlling pain at this level or iii) a minimal threshold concentration of EP1 receptor-associated transduction enzyme despite the elevated number of EP1 receptors in the VL PAG in normal conditions. Further studies are necessary to clarify this issue.

Prostaglandin-induced pain facilitation involves the recruitment of the pain-responding neuron population in the RVM [11]. The PAG modulatory effect on pain control involves RVM neuron activity. In this study, intra-VL PAG ONO-DI-004 modified both spontaneous and tail flick-related activities of ON and OFF cells. In particular, it increased the spontaneous activity of the "pronociceptive" ON cells and reduced the spontaneous activity of the "antinociceptive" OFF cells, as a facilitating nociception drug is expected to do [11]. ONO-DI-004 also decreased the onset of the ON cell burst and OFF cell pause, together with an increase in the burst frequency and pause duration; all of which are effects that are critical for pain facilitation. The same effects on RVM cell activity were produced by PGE2, which stimulates all EP1-4 receptors. In our study we also observed that the effect of ONO-DI-004, and more intriguingly that of PGE2, were completely prevented by L335677, a selective EP1 receptor antagonist. PGE2 has a high affinity for all EP receptors [36], thus the complete blockade of PGE2-induced behavioural and electrophysiological effects by L335677 would suggest that: i) the participation of all EP receptors is necessary for the nociceptive facilitation as in "an in series" circuitry; ii) receptors other than EP1 and EP3 are not present within the VL PAG, a hypothesis which is however contradicted by a previous study [12] and iii) EP2, EP3 and EP4 receptors are not involved in controlling nociceptive and ON and OFF cell activity responses. The most likely possibility in our opinion would seem to be that the involvement of all EP receptors is required for the nociceptive facilitation of PGE2, since apart from preventing misoprostol-induced hyperalgesia [12] within the DL PAG, individual EP1, EP2, EP3 and EP4 receptor antagonists also proved to be analgesic in formalin-induced persistent pain [12]. We cannot exclude, however, that in the VL PAG sub-region, in healthy animals and in a different rodent species (rats versus mice) EP2, EP3 and EP4 are not involved in controlling nociception or RVM cell activity, or that the higher doses used in one of our previous studies [12] have involved other receptor subtypes. Further studies with selective EP2, EP3 and EP4 receptor antagonists or knock out mice are therefore necessary in order to clarify this issue. However, what seems important in the current study is that intra-VL PAG microinjection of L335677 inhibits nociception and reduces the spontaneous and tail flick-related activity of the ON cells in the RVM. Moreover, L335677 produced an increase in spontaneous OFF cell activity as well as an increase in the onset of the OFF cell pause, together with a decrease in the duration of the pause. Such effects are consistent with behavioural analgesia and could be important in experimental pain models such as inflammatory and neuropathic pain.

Our data shows that 7 days after sciatic nerve insult, RVM cell activity changes in such a way that the ongoing ON cell activity increased and that of the OFF cell decreased. Apart from the ongoing activity, the tail flick stimulated activity also changed in nerve injured rats. The burst and onset of the burst of the ON cells increased and decreased, respectively. Consistently, the pause and onset of the pause of the OFF cell increased and decreased respectively in neuropathic rats. Thus it appears that ON and OFF cell activity in the RVM undergoes functional phenotypic change after SNI which leads to ON cell hyperactivity and OFF cell hypoactivity after neuropathic pain induction. Changes in the pain descending system contributing to chronic pain symptoms have been reported [37-40]. In particular an increased activity of ON cell, together with a depression of OFF cell activity 1 week after SNI have already been observed within the RVM [41]. Moreover in this study, as well as decreasing tail flick latency EP1 receptor stimulation was still able to increase and decrease ON and OFF cell activities as far as the ongoing or the flick related activity was concerned. More interestingly, EP1 receptor blockade by L335677, at the same doses used in healthy animals, increased tail flick latency and modified the ongoing and tail flick-evoked activity of ON and OFF cells, consistently with behavioural analgesia. Evidence of effectiveness of a selective EP1 receptor antagonist on hyperalgesia and allodynia in neuropathic pain state has already been reported [17,42] after systemic administration.

The first part of the study showed the location of EP1 receptor within VGAT positive cell population within the VL PAG. EP1 receptor stimulation is associated with changes in calcium concentration and neurotransmitter release, which would increase GABAergic tone, thereby inhibiting the antinociceptive descending pathway. This would generate behavioural pain facilitation through a GABAergic interneuron activation of RVM ON cells and a direct inhibition of RVM OFF cells. Conversely, the tonically active EP1 receptor blockade would generate the opposite effect and dis-inhibit the PAG descending pathway, generating antinociception. EP1 receptor blockade-induced nociception inhibition at PAG level is associated with ON cell inhibition and OFF cell activation (and with the opposite electrophysiological effect in the case of ONO-DI-004 and PGE2). As far as the RVM ON and OFF cell involvement on intra-VL PAG ONO-DI-004, PGE2 and L335677 effect is concerned, the current study only shows correlative and synchronous changes in ON and OFF cell activity and thermonociceptive responses. Nevertheless there is increasing evidence to suggest that the switching off of the ON cells, which are the nociceptive facilitating neurons to the dorsal horns [43,44] and activation of OFF cell activity are critical events in the production of antinociception. Another issue of the study regards the doses of ONO-DI-004, L335677 and PGE2 chosen. As far as the PGE2 is concerned, the doses used in the current study match those used in another study using the same intra-PAG administration route [11]. ONO-DI-004 and L335667 have never been microinjected within the PAG. Therefore given that most synthetic EP receptor compounds mantain selectivity for their target receptor subtype in low nM, or an even lower range, we microinjected doses (few pgs in 0.2 μl) which are within or even under the limit of selectivity. Moreover, we tested for both L335677 (0.1 pg) and ONO-DI-004 (0.001 pg) tenfold lower doses, which proved to be devoid of activity.

## Conclusions

In conclusion, this study suggests that the presence within the VL PAG of EP1 receptor, whose activation with a selective EP1 receptor agonist ONO-DI-004 or PGE2, which stimulates all EP receptors, facilitates nociceptive responses and modifies the activity of RVM ON and OFF cells, consistently with pain facilitation, may be a suitable target for inducing antinociception. Indeed, its blockade by L335677, a selective EP1 receptor antagonist, leads to pain inhibition through the depression and enhancement of ON and OFF cell activity, respectively, in normal and neuropathic pain conditions. Although VL PAG EP1 receptor shows approximately 50% loss of expression in neuropathic conditions, EP1 receptor blockade still alleviates pain response. The mechanism by which EP1 ligands maintain their efficacy in the presence of EP1 downregulation in neuropathic pain warrants further study.

## Abbreviations

(VL PAG): ventrolateral periaqueductal grey; (RVM): rostral ventromedial medulla; (PGs): prostaglandins; (COXs): cyclooxygenases; (SNI) spared nerve injury; (VGluT1): anti-vesicular glutamate transporter-1; (VGAT): anti-vesicular GABA transporter; (DMSO): dimethyl sulfoxide; (ACSF): artificial cerebrospinal fluid; (PGE2): prostaglandin E2: (L-335677): (5Z: 11α: 13E: 15S)-11: 15-dihydroxy-9-oxo-prosta-5: 13-dien1oic acid.

## Competing interests

The authors declare that they have no competing interests.

## Authors' contributions

EP has written the manuscript. EP and SM have conceived and conceptualized the manuscript. FG, LG and SB have carried out the electrophysiological experiments. LL has contributed with immunohistochemistry. GB has performed western blot experiment and analysis. IM, VdN and FR contributed to the drafting of the paper. All authors have read and approved the final manuscript.
